# The Predictive Role of NLR, d-NLR, MLR, and SIRI in COVID-19 Mortality

**DOI:** 10.3390/diagnostics12010122

**Published:** 2022-01-06

**Authors:** Cosmin Citu, Florin Gorun, Andrei Motoc, Ioan Sas, Oana Maria Gorun, Bogdan Burlea, Ioana Tuta-Sas, Larisa Tomescu, Radu Neamtu, Daniel Malita, Ioana Mihaela Citu

**Affiliations:** 1 Department of Obstetrics and Gynecology, “Victor Babes” University of Medicine and Pharmacy Timisoara, 2 Eftimie Murgu Square, 300041 Timisoara, Romania; citu.ioan@umft.ro (C.C.); sasioan56@yahoo.com (I.S.); tomescu.larisa@umft.ro (L.T.); radu.neamtu@umft.ro (R.N.); 2 Department of Anatomy and Embryology, “Victor Babes” University of Medicine and Pharmacy Timisoara, 2 Eftimie Murgu Square, 300041 Timisoara, Romania; amotoc@umft.ro; 3 Department of Obstetrics and Gynecology, Municipal Emergency Clinical Hospital Timisoara, 1–3 Alexandru Odobescu Street, 300202 Timisoara, Romania; oanabalan@hotmail.com (O.M.G.); bogdanburlea@yahoo.com (B.B.); 4 Discipline of Hygiene, Department 14 Microbiology, “Victor Babes” University of Medicine and Pharmacy Timisoara, 2 Eftimie Murgu Square, 300041 Timisoara, Romania; tuta-sas.ioana@umft.ro; 5 Department of Radiology, “Victor Babes” University of Medicine and Pharmacy Timisoara, Eftimie Murgu Square nr. 2, 300041 Timisoara, Romania; malita.daniel@umft.ro; 6 Department of Internal Medicine I, “Victor Babes” University of Medicine and Pharmacy Timisoara, 2 Eftimie Murgu Square, 300041 Timisoara, Romania; citu.ioana@umft.ro

**Keywords:** COVID-19, predictive, inflammation, mortality

## Abstract

(1) Background: Since its discovery, COVID-19 has caused more than 256 million cases, with a cumulative death toll of more than 5.1 million, worldwide. Early identification of patients at high risk of mortality is of great importance in saving the lives of COVID-19 patients. The study aims to assess the utility of various inflammatory markers in predicting mortality among hospitalized patients with COVID-19. (2) Methods: A retrospective observational study was conducted among 108 patients with laboratory-confirmed COVID-19 hospitalized between 1 May 2021 and 31 October 2021 at Municipal Emergency Clinical Hospital of Timisoara, Romania. Blood cell counts at admission were used to obtain NLR, dNLR, MLR, PLR, SII, and SIRI. The association of inflammatory index and mortality was assessed via Kaplan–Maier curves univariate Cox regression and binominal logistic regression. (3) Results: The median age was 63.31 ± 14.83, the rate of in-hospital death being 15.7%. The optimal cutoff for NLR, dNLR, MLR, and SIRI was 9.1, 9.6, 0.69, and 2.2. AUC for PLR and SII had no statistically significant discriminatory value. The binary logistic regression identified elevated NLR (aOR = 4.14), dNLR (aOR = 14.09), and MLR (aOR = 3.29), as independent factors for poor clinical outcome of COVID-19. (4) Conclusions: NLR, dNLR, MLR have significant predictive value in COVID-19 mortality.

## 1. Introduction

In December 2019, in Wuhan, Hubei Province, China reported cases of pneumonia with an unknown virus [1]. Later, on 11 February 2020, ICTV (International Committee on Taxonomy of Viruses) announced “severe acute respiratory syndrome coronavirus 2 (SARS-CoV-2)” as the name of the new virus, and the disease was named by WHO (World Health Organization) as “COVID-19” [2]. Due to a huge increase in the number of cases, on 11 March 2020, WHO has officially declared a pandemic of COVID-19 [3]. Since its discovery, the virus has caused more than 256 million cases, with a cumulative death toll of more than 5.1 million, worldwide [4]. In Romania, more than 1.7 million confirmed cases of COVID-19 and more than 56,000 deaths have been reported by December 2021, considering that the first confirmed case of COVID-19 in Romania was on 26 February 2020 [5,6]. The main method by which COVID-19 is diagnosed is the detection of nucleic acid by real-time polymerase chain reaction (RT-PCR) [7,8]. COVID-19 is an inflammatory disease caused by SARS-CoV-2 and can manifest as various symptoms ranging from mild symptoms or asymptomatic cases to severe pneumonia that can progress to acute respiratory distress syndrome (ARDS) and death [9]. This disease has a three-phase progression: initial disease caused by active infection, a second pulmonary phase, and, when severe, a third phase described by hyper-inflammation, cytokine storm, elevated biomarker levels of cardiac injury, and significant morbidity and mortality [10]. Serum biochemical analysis and blood count analysis are commonly used blood tests, which could be faster, easier to use, and low-cost techniques that can facilitate the diagnosis and prognosis of this disease [11]. From these routine tests, inflammatory markers have been used for predicting the severity of COVID-19 such as neutrophil to lymphocyte ratio (NLR), derivate neutrophil to lymphocyte ratio (dNLR), monocyte to lymphocyte ratio (MLR), and platelet to lymphocyte ratio (PLR) [11,12]. NLR and PLR are biomarkers reflecting systemic inflammation, neutrophil and platelet activation, and are associated with increased mortality in cardiovascular disease and poor prognosis in various cancers or in polycythemia vera [13,14,15]. In addition, a higher NLR, and decreased PLR were predictive of poor survival in patients with myelofibrosis [16]. Derived neutrophil-to-lymphocyte ratio (dNLR) is a potential new biomarker for systemic inflammation, defined as the absolute neutrophil count (ANC)/white cell count (WBC)—absolute neutrophil count (ANC), and has prognostic value in patients with several types of cancer [17,18,19]. Unlike NLR, dNLR includes monocytes and other granulocytes by using the difference between WBC and neutrophils in the denominator. Poorly differentiated and immature neutrophils can be released into a pro-inflammatory environment, which rapidly increases neutrophil generation, thus dNLR is likely to reflect this negative inflammation more comprehensively [17,19]. The systemic inflammatory response index (SIRI) may also reflect the host’s immune and inflammatory balance [20]. In addition, systemic immune-inflammation index (SII), defined as platelet count × NLR, is effective in reflecting inflammatory status, being a basic biomarker for predicting the prognosis [12]. The current study assesses the utility of various inflammatory markers in predicting mortality among hospitalized patients with COVID-19. 

## 2. Materials and Methods

### 2.1. Study Design

A single-center retrospective observational study was conducted to assess inflammatory biomarkers as prognostic for complications of COVID-19. This study was conducted on patients admitted with COVID-19 at the Municipal Emergency Clinical Hospital of Timisoara, Romania, between 1 May 2021 and 31 October 2021. The study was approved by the Ethics Committee of the University of Medicine and Pharmacy “Victor Babes” Timisoara (No. 22726/17 November 2021).

### 2.2. Participants

Patients enrolled in the study met the following criteria: (1) diagnosed with COVID-19 according to the guidelines issued by the National Center for Surveillance and Control of Communicable Diseases Romania, being tested positive for SARS-CoV-2 using real-time reverse transcriptase-polymerase chain reaction (RT-PCR) on a nasopharyngeal swab; (2) hospital admission from 1 May 2021, and 31 October 2021; (3) documented complete blood count; (4) age over 18 years. Patients under 18 years old or having missing laboratory data were excluded.

### 2.3. Variables, Data Sources, and Measurement

Data were extracted by three researchers from patients’ electronic medical records, using a standardized data collection form. Demographic elements, clinical data, and laboratory assessments collected were age, sex, and comorbidities, complete blood count on hospital admission, oxygen saturation in room air on admission, and length of hospitalization or days until hospital death. Systemic inflammation indexes were determined from the initial complete blood count using the following formulas: NLR = absolute neutrophil count (ANC)/absolute lymphocyte count (ALC); dNLR = ANC/(WBC − ANC); MLR = absolute monocyte count divided/ALC; PLR = absolute platelet count (APC)/ALC; SII = (ANC × APC)/ALC; SIRI = (ANC × AMC)/ALC). The outcome of interest was in-hospital mortality.

### 2.4. Statistical Analysis

Statistical calculations were performed using SPSS 20.0 software (SPSS Inc., Chicago, IL, USA). Continuous variables were expressed as mean ± standard deviation and the comparison between them was performed using independent sample *t*-test. Categorical variables were expressed in count and percentage and were compared using Fisher’s exact test. The predictive performance of the indexes for death was assessed by estimating the area under the curve and the corresponding of the receiver operating characteristic curve method. The optimal cutoff values of inflammatory indexes were determined using the Youden’s index. The association was estimated by a univariate Cox proportional hazards model and binominal logistic regression.

## 3. Results

### 3.1. Participants Characteristics

A total of 108 patients diagnosed with COVID-19 were enrolled in the study and followed up during hospitalization. The average hospitalization duration was 11.89 (SD: 6.56) days. The mean (SD) age of patients was 63.31 (14.83) years, and more than half were men (51.9%). The overall number of in-hospital deaths was 17 (15.7%). Compared to patients in the survivor cohort, in-hospital dead patients were significantly older, but no significant difference was observed in terms of gender. The most common comorbidity was hypertension (70.4%), followed by other heart diseases (47.2%) diabetes (46.3%), and chronic lung disease (21.3%). Except for heart disease, there was no statistically significant difference in the comorbidity frequency between surviving and dead patients. The laboratory data of all patients on admission are shown in Table 1. Several variables were significantly associated with poor outcomes, dead patients had lower lymphocyte and platelet counts, and higher NLR, dNLR, MLR, and SIRI.

### 3.2. Using Optimal Cut-Off Values of Inflammatory Markers to Predict Mortality in Patients with COVID-19

**Figure 1:**
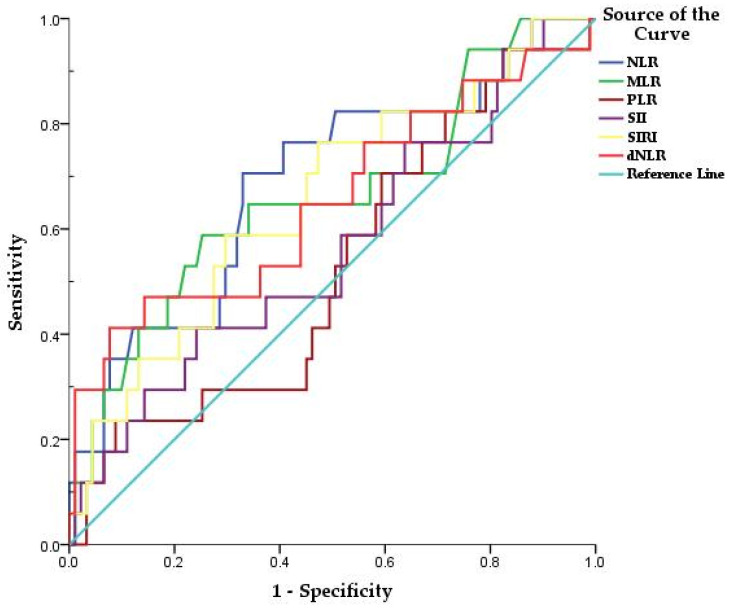
Receiver operating characteristic (ROC) curves of NLR, DNLR, MLR, PLR, SII, and SIRI in predicting death, in patients with COVID-19.

Receiver operating characteristic (ROC) curves of NLR, dNLR, MLR, PLR, SII, and SIRI were created to determine whether the baseline of these biomarkers was predictive mortality in patients with COVID-19 (Figure 1). The areas under the curve (AUC) of NLR, dNLR, MLR, SIRI were above 0.6 (Table 2). The optimal cutoff obtained from Youden’s index is listed in Table 2. PLR and SII had AUC <0.6 (0.525 and 0.564 respectively) and no statistical significance (*p* > 0.05) being excluded.

### 3.3. Association of Inflammatory Biomarkers Results with The COVID-19 Mortality

**Figure 2:**
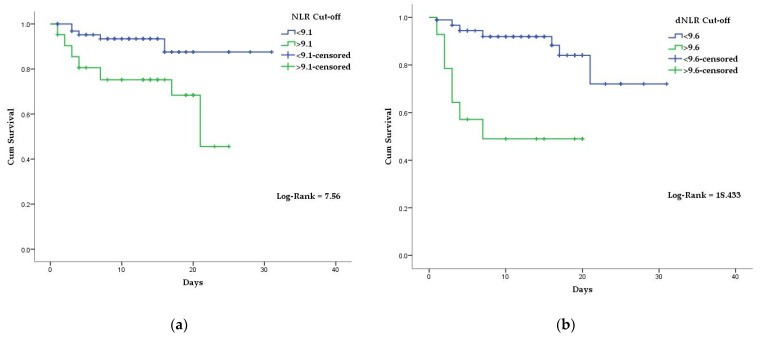
Kaplan–Meier survival curves of hospitalized COVID-19 patients: (**a**) according to established NLR cutoff values; (**b**) according to established dNLR cutoff values.

**Figure 3:**
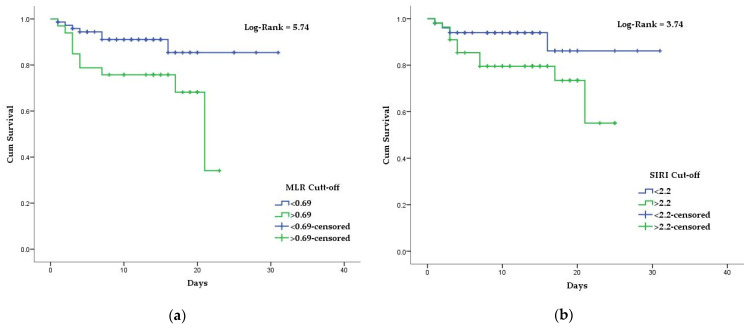
Kaplan–Meier survival curves of hospitalized COVID-19 patients: (**a**) according to established MLR cutoff values; (**b**) according to established SIRI cutoff values.

Kaplan–Meier curves and the univariate Cox regression model were created, using the established NLR, dNLR, MLR, and SIRI cutoff points. Mean survival time for COVID-19 patients above the stated NLR, dNLR, MLR, and SIRI cutoff values were 18.2 days, 11.4 days, 17.1 days, and 19.5 days, respectively. In comparison, the mean survival time for COVID-19 patients with bellow the stated NLR, dNLR, MLR, and SIRI cutoff values were 28.3 days, 26.5 days, 27.7 days, and 28.0 days, respectively (Figure 2 and Figure 3). Differences in survival for patients above the baseline reported NLR, dNLR, and MLR compared to those below the baseline were highly statistically significant (*p* < 0.001 for each). However, the differences in survival for patients with SIRI values above the stated cutoff compared to those below the cutoff were not statistically significant (*p* = 0.05).

Furthermore, univariate Cox regression analysis showed that NLR, dNLR, and MLR were independent predictors of in-hospital mortality (Table 3).

**Figure 4:**
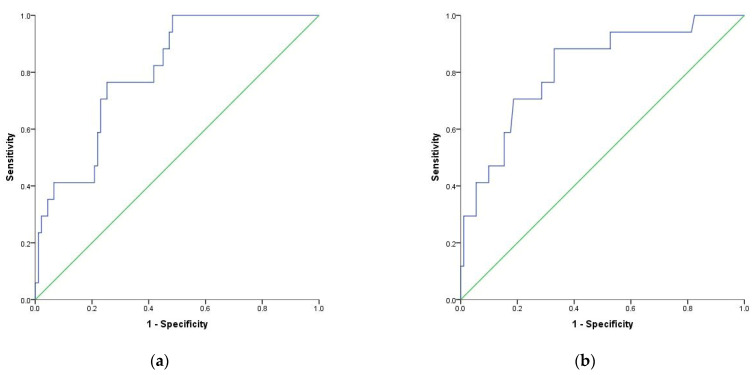
Receiver operating characteristic (ROC) curve for logistic regression models: (**a**) NLR (above or below 9.1) as a prognostic factor of mortality adjusted to age, sex and comorbidities index; (**b**) dNLR (above or below 9.6) as a prognostic factor of mortality adjusted to age, sex and comorbidities.

**Figure 5:**
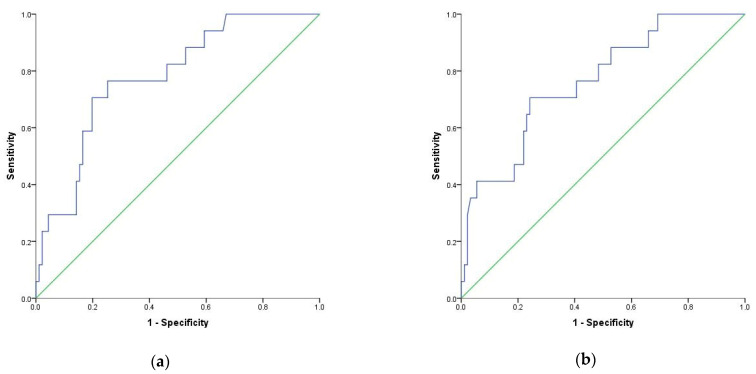
Receiver operating characteristic (ROC) curve for logistic regression models: (**a**) MLR (above or below 0.69) as a prognostic factor of mortality adjusted to age, sex and comorbidities index; (**b**) SIRI (above or below 2.2) as a prognostic factor of mortality adjusted to age, sex and comorbidities.

Additionally, multivariate logistic regression was performed to test the discrimination ability of NLR, dNLR, MLR, and SIRI (above or below cutoff values) as prognostics factors of mortality adjusted for age, comorbidities, COVID-19 severity, and sex. Results showed an aOR of 4.77 for NLR above 9.1 of 14.09 for dNLR above 9.6 of 3.29 for MLR above 0.69 and 3.06 for SIRI above 2.2 (Table 4). The AUC for logistic regression models used are 0.788, 0.812, 0.779, and 0.763, respectively (Figure 4 and Figure 5).

## 4. Discussion

This study reported data from 108 patients hospitalized with COVID-19 at the Municipal Emergency Clinical Hospital Timisoara, Romania. Of these, 17 (15.7%) patients died during hospitalization. COVID-19 is a more severe respiratory illness than seasonal flu, resulting in about 5% of patients diagnosed with this condition requiring intensive care hospitalization and about 3% dying [21]. In addition, Ana Macedo et al., showed in a systematic review an overall 17% mortality rate for COVID-19 patients admitted to hospitals [22].

Demographic data from our study show that COVID-19 patients who died were significantly older compared to survivors. Similar to our results, several reports from previous studies showed that disease severity was significantly related to age [23,24,25]. However, a study conducted in Italy showed no age differences between COVID-19 patients admitted to ICU compared to the general positive population suggesting that age alone is not a risk factor for ICU admission [26].

However, contrary to those found among patients in our cohort, several recent studies have demonstrated that the presence of comorbidities, such as hypertension, diabetes, respiratory disease, or cardiovascular disease, each increased the risk of progression to severe illness [23,24,27]. In our study, only heart disease was associated with COVID-19 mortality in univariate analysis.

Regarding laboratory tests, several abnormalities have been reported in patients with COVID-19, leukocytosis, lymphopenia, and increased neutrophil count being related to disease severity [25,28,29,30]. Our data show no significant difference in mean leukocyte, lymphocyte or neutrophil concentrations between surviving and dead patients.

However, many reports have identified NLR, dNLR, MLR as independent risk factors for severe disease [8,11,25,28,31,32].

In our study, the cutoff points for NLR, dNLR, MLR and SIRI were observed using the ROC curve. The optimal thresholds of 9.1, 9.6, 0.69, and 2.2 for NLR, dNLR, MLR, and SIRI, respectively, showed a superior prognostic possibility for mortality with the highest sensitivity and specificity on AUC.

NLR is related to systemic inflammatory status and disease activity and has prognostic value in cardiovascular disease, autoimmune disease, tumors, and other infectious diseases [33,34,35]. NLR is also included as a variable in a risk score to predict the occurrence of critical illness in patients with COVID-19, and some studies have identified its role in discriminating severe disease and predicting mortality [8,11,25,31,32,36,37,38].

The results of the present study are concordant with the findings of these studies, with NLR, dNLR, and MLR being predictors of mortality in patients with COVID-19, with HR of 3.85, 6.4, and 3.05 on univariate Cox regression. Additionally, multivariate analysis showed an adjusted OR of 4.14, 14.09, 3.29, 3.06 for NLR, dNLR, MLR, and SIRI. However, contrary to what has been shown in other studies, in our study the AUC for PLR and SII had no statistically significant discriminatory value. PLR has been used as a predictor in various diseases, such as cardiovascular and autoimmune diseases [39]. In addition, PLR on admission was found to be higher in severe COVID-19 compared to non-severe cases, in some studies [39]. Furthermore, two studies found significant correlations of SII in predicting mortality, being even superior to NLR or dNLR [25,40]. In addition, SIRI did not show a statistically significant predictive value of COVID-19 mortality in our study, contrary to those shown in other studies [25].

This paper has several limitations. First, the study has a retrospective design, and the data were obtained from a single clinic. Second, the sample may not have been large enough to assess the predictive performance of NLR, dNLR, MLR, PLR, SIRI, and SII for death, as only 17 deaths were included in the analysis in this cohort. Furthermore, we could not exclude the impact of some treatments before hospital admission on the outcome of NLR, dNLR, MLR, PLR SIRI, and SII.

In conclusion, in this retrospective cohort study, NLR, dNLR, MLR determined at hospital admission had a high value in predicting death among patients with COVID-19. Future clinical research efforts should examine strategies to reduce the effects associated with elevated levels of these indexes in order to improve treatment and reduce mortality.

## Figures and Tables

**Table 1:** Baseline characteristics and laboratory test results in 108 hospitalized patients with COVID-19.

	Total	Survivors 91 (84.3%)	Deaths 17 (15.7%)	*p* Value
**Age** (Mean ± SD)	63.31 ± 14.83	62.02 ± 14.73	70.18 ± 13.83	0.03
**Comorbidities** No. (%)				
Diabetes	50 (46.3%)	40 (44.0%)	10 (58.8%)	0.29
Hypertension	76 (70.4%)	62 (68.1%)	14 (82.4%)	0.38
Heart diseases	51 (47.2%)	38 (41.8%)	13 (76.5%)	0.01
Chronic lung diseases	23 (21.3%)	17 (18.7%)	6 (35.3%)	0.19
**Complete blood count** (Mean ± SD)				
White blood cell (×10^12^/L)	8.71 ± 5.74	8.71 ± 5.76	8.73 ± 5.81	0.98
Neutrophil count (×10^9^/L)	6.96 ± 4.36	6.75 ± 4.18	8.06 ± 5.19	0.25
Lymphocyte count (×10^9^/L)	0.98 ± 0.78	1.03 ± 0.82	0.73 ± 0.44	0.03
Monocyte count (×10^9^/L)	0.47 ± 0.32	0.47 ± 0.32	0.51 ± 0.33	0.64
Hemoglobin (g/dL)	13.15 ± 1.78	13.27 ± 1.64	12.50 ± 2.36	0.10
Platelet count (×10^9^/L)	242 ± 109	252 ± 112	192 ± 79	0.03
**Inflammatory markers**				
NLR	9.18 ± 6.7	8.31 ± 5.74	13.83 ± 9.23	0.001
MLR	0.58 ±0.44	0.53 ± 0.39	0.83 ± 0.59	0.01
PLR	327 ± 72	324 ± 219	345 ± 235	0.71
dNLR	5.16 ± 3.76	4.77 ± 3.45	7.07 ± 4.64	0.01
SII	2280 ± 1950	2183 ± 1847	2798.± 2429	0.23
SIRI	4.57 ± 5.12	4.11 ± 4.67	7.02 ± 6.72	0.03
O_2_ Saturation *	91.96 ± 6.16	92.26 ± 5.96	90.35 ± 7.13	0.24
**Hospitalization length**	11.89 (6.56)	12.96	6.18	<0.001

* Room air oxygen saturation levels

**Table 2:** Receiver operating characteristics (ROC) curves, prognostic accuracy of inflammatory markers, and optimal cutoff.

Variables	Area	Std. Error	Asymptotic Sig.	95% Confidence Interval		Sensitivity	Sensibility	Cut-Off
				Lower	Upper			
NLR	0.689	0.074	0.014	0.544	0.833	70%	67%	9.1
MLR	0.661	0.078	0.036	0.508	0.813	58%	74%	0.69
SIRI	0.655	0.074	0.042	0.511	0.800	76%	52%	2.2
dNLR	0.652	0.082	0.047	0.491	0.813	41%	92%	9.6

**Table 3:** Hazard ratios of the indexes obtained by univariate Cox regression analysis.

Variables	HR (95%CI)	*p* Value
NLR	3.85 (1.35–10.95)	0.01
dNLR	6.4 (2.40–17.18)	<0.001
MLR	3.05 (1.16–8.05)	0.02

**Table 4:** The adjusted OR in each of the NLR, d-NLR, MLR and SIRI.

Variables	Adjusted OR *	*p* Value
NLR	4.14	0.002
dNLR	14.09	0.001
MLR	3.29	0.04
SIRI	3.06	0.08

* Adjustment for age, comorbidities, and sex. Each of NLR, MLR, dNLR, and SIRI were included in four different models for aOR calculation.

## Data Availability

The data sets used and/or analyzed during the present study are available from the correspondence author on reasonable request.

## References

[1] Zhu N., Zhang D., Wang W., Li X., Yang B., Song J., Zhao X., Huang B., Shi W., Lu R. (2020). A Novel Coronavirus from Patients with Pneumonia in China, 2019. N. Engl. J. Med..

[2] Naming the Coronavirus Disease (COVID-19) and the Virus That Causes It. https://www.who.int/emergencies/diseases/novel-coronavirus-2019/technical-guidance/naming-the-coronavirus-disease-(covid-2019)-and-the-virus-that-causes-it.

[3] Cucinotta D., Vanelli M. (2020). WHO Declares COVID-19 a Pandemic. Acta Bio Med. Atenei Parm..

[4] Weekly Epidemiological Update on COVID-19—23 November 2021. https://www.who.int/publications/m/item/weekly-epidemiological-update-on-covid-19---23-november-2021.

[5] Ritchie H., Mathieu E., Rodés-Guirao L., Appel C., Giattino C., Ortiz-Ospina E., Hasell J., Macdonald B., Beltekian D., Roser M. Coronavirus Pandemic (COVID-19). https://ourworldindata.org/coronavirus.

[6] Dascalu S. (2020). The Successes and Failures of the Initial COVID-19 Pandemic Response in Romania. Front. Public Health.

[7] Alsharif W., Qurashi A. (2021). Effectiveness of COVID-19 Diagnosis and Management Tools: A Review. Radiography.

[8] Seyit M., Avci E., Nar R., Senol H., Yilmaz A., Ozen M., Oskay A., Aybek H. (2021). Neutrophil to Lymphocyte Ratio, Lymphocyte to Monocyte Ratio and Platelet to Lymphocyte Ratio to Predict the Severity of COVID-19. Am. J. Emerg. Med..

[9] Rodrigues T.S., de Sá K.S.G., Ishimoto A.Y., Becerra A., Oliveira S., Almeida L., Gonçalves A.V., Perucello D.B., Andrade W.A., Castro R. (2021). Inflammasomes Are Activated in Response to SARS-CoV-2 Infection and Are Associated with COVID-19 Severity in Patients. J. Exp. Med..

[10] Koupenova M., Freedman J.E. (2020). Platelets and COVID-19: Inflammation, Hyperactivation and Additional Questions. Circ. Res..

[11] Yang A.-P., Liu J., Tao W., Li H. (2020). The Diagnostic and Predictive Role of NLR, d-NLR and PLR in COVID-19 Patients. Int. Immunopharmacol..

[12] Karimi A., Shobeiri P., Kulasinghe A., Rezaei N. (2021). Novel Systemic Inflammation Markers to Predict COVID-19 Prognosis. Front. Immunol..

[13] Hirahara T., Arigami T., Yanagita S., Matsushita D., Uchikado Y., Kita Y., Mori S., Sasaki K., Omoto I., Kurahara H. (2019). Combined Neutrophil-Lymphocyte Ratio and Platelet-Lymphocyte Ratio Predicts Chemotherapy Response and Prognosis in Patients with Advanced Gastric Cancer. BMC Cancer.

[14] Zhang Y., Lu J.-J., Du Y.-P., Feng C.-X., Wang L.-Q., Chen M.-B. (2018). Prognostic Value of Neutrophil-to-Lymphocyte Ratio and Platelet-to-Lymphocyte Ratio in Gastric Cancer. Medicine.

[15] Krečak I., Holik H., Morić Perić M., Zekanović I., Coha B., Valovičić Krečak M., Gverić-Krečak V., Lucijanić M. (2021). Neutrophil-to-lymphocyte and Platelet-to-lymphocyte Ratios as Prognostic Biomarkers in Polycythemia Vera [Online early access]. Int. J. Lab. Hematol..

[16] Lucijanic M., Cicic D., Stoos-Veic T., Pejsa V., Lucijanic J., Fazlic Dzankic A., Vlasac Glasnovic J., Soric E., Skelin M., Kusec R. (2018). Elevated Neutrophil-to-Lymphocyte-Ratio and Platelet-to-Lymphocyte Ratio in Myelofibrosis: Inflammatory Biomarkers or Representatives of Myeloproliferation Itself?. Anticancer Res..

[17] Proctor M.J., McMillan D.C., Morrison D.S., Fletcher C.D., Horgan P.G., Clarke S.J. (2012). A Derived Neutrophil to Lymphocyte Ratio Predicts Survival in Patients with Cancer. Br. J. Cancer.

[18] Grenader T., Nash S., Adams R., Kaplan R., Fisher D., Maughan T., Bridgewater J. (2016). Derived Neutrophil Lymphocyte Ratio Is Predictive of Survival from Intermittent Therapy in Advanced Colorectal Cancer: A Post Hoc Analysis of the MRC COIN Study. Br. J. Cancer.

[19] Yang T., Hao L., Yang X., Luo C., Wang G., Cai C.L., Qi S., Li Z. (2021). Prognostic Value of Derived Neutrophil-to-lymphocyte Ratio (DNLR) in Patients with Non-small Cell Lung Cancer Receiving Immune Checkpoint Inhibitors: A Meta-analysis. BMJ Open.

[20] Jiang S., Wang S., Wang Q., Deng C., Feng Y., Ma F., Ma J., Liu X., Hu C., Hou T. (2021). Systemic Inflammation Response Index (SIRI) Independently Predicts Survival in Advanced Lung Adenocarcinoma Patients Treated with First-Generation EGFR-TKIs. Cancer Manag. Res..

[21] Citu C., Neamtu R., Sorop V.-B., Horhat D.I., Gorun F., Tudorache E., Gorun O.M., Boarta A., Tuta-Sas I., Citu I.M. (2021). Assessing SARS-CoV-2 Vertical Transmission and Neonatal Complications. J. Clin. Med..

[22] Macedo A., Gonçalves N., Febra C. (2021). COVID-19 Fatality Rates in Hospitalized Patients: Systematic Review and Meta-Analysis. Ann. Epidemiol..

[23] Zhou F., Yu T., Du R., Fan G., Liu Y., Liu Z., Xiang J., Wang Y., Song B., Gu X. (2020). Clinical Course and Risk Factors for Mortality of Adult Inpatients with COVID-19 in Wuhan, China: A Retrospective Cohort Study. Lancet.

[24] Chen L., Yu J., He W., Chen L., Yuan G., Dong F., Chen W., Cao Y., Yang J., Cai L. (2020). Risk Factors for Death in 1859 Subjects with COVID-19. Leukemia.

[25] Fois A.G., Paliogiannis P., Scano V., Cau S., Babudieri S., Perra R., Ruzzittu G., Zinellu E., Pirina P., Carru C. (2020). The Systemic Inflammation Index on Admission Predicts In-Hospital Mortality in COVID-19 Patients. Molecules.

[26] Grasselli G., Zangrillo A., Zanella A., Antonelli M., Cabrini L., Castelli A., Cereda D., Coluccello A., Foti G., Fumagalli R. (2020). Baseline Characteristics and Outcomes of 1591 Patients Infected With SARS-CoV-2 Admitted to ICUs of the Lombardy Region, Italy. JAMA.

[27] Yang J., Zheng Y., Gou X., Pu K., Chen Z., Guo Q., Ji R., Wang H., Wang Y., Zhou Y. (2020). Prevalence of Comorbidities and Its Effects in Patients Infected with SARS-CoV-2: A Systematic Review and Meta-Analysis. Int. J. Infect. Dis..

[28] Huang C., Wang Y., Li X., Ren L., Zhao J., Hu Y., Zhang L., Fan G., Xu J., Gu X. (2020). Clinical Features of Patients Infected with 2019 Novel Coronavirus in Wuhan, China. Lancet.

[29] Gong J., Ou J., Qiu X., Jie Y., Chen Y., Yuan L., Cao J., Tan M., Xu W., Zheng F. (2020). A Tool for Early Prediction of Severe Coronavirus Disease 2019 (COVID-19): A Multicenter Study Using the Risk Nomogram in Wuhan and Guangdong, China. Clin. Infect. Dis..

[30] Itelman E., Wasserstrum Y., Segev A., Avaky C., Negru L., Cohen D., Turpashvili N., Anani S., Zilber E., Lasman N. (2020). Clinical Characterization of 162 COVID-19 Patients in Israel: Preliminary Report from a Large Tertiary Center. Isr. Med. Assoc. J..

[31] Tatum D., Taghavi S., Houghton A., Stover J., Toraih E., Duchesne J. (2020). Neutrophil-to-Lymphocyte Ratio and Outcomes in Louisiana COVID-19 Patients. Shock Augusta Ga.

[32] Prozan L., Shusterman E., Ablin J., Mitelpunkt A., Weiss-Meilik A., Adler A., Choshen G., Kehat O. (2021). Prognostic Value of Neutrophil-to-Lymphocyte Ratio in COVID-19 Compared with Influenza and Respiratory Syncytial Virus Infection. Sci. Rep..

[33] Huguet E., Maccallini G., Pardini P., Hidalgo M., Obregon S., Botto F., Koretzky M., Nilsson P.M., Ferdinand K., Kotliar C. (2021). Reference Values for Neutrophil to Lymphocyte Ratio (NLR), a Biomarker of Cardiovascular Risk, According to Age and Sex in a Latin American Population. Curr. Probl. Cardiol..

[34] Wang X., Qiu L., Li Z., Wang X.-Y., Yi H. (2018). Understanding the Multifaceted Role of Neutrophils in Cancer and Autoimmune Diseases. Front. Immunol..

[35] Zeng Z.-Y., Feng S.-D., Chen G.-P., Wu J.-N. (2021). Predictive Value of the Neutrophil to Lymphocyte Ratio for Disease Deterioration and Serious Adverse Outcomes in Patients with COVID-19: A Prospective Cohort Study. BMC Infect. Dis..

[36] Liang W., Liang H., Ou L., Chen B., Chen A., Li C., Li Y., Guan W., Sang L., Lu J. (2020). Development and Validation of a Clinical Risk Score to Predict the Occurrence of Critical Illness in Hospitalized Patients With COVID-19. JAMA Intern. Med..

[37] Liu Y., Du X., Chen J., Jin Y., Peng L., Wang H.H.X., Luo M., Chen L., Zhao Y. (2020). Neutrophil-to-Lymphocyte Ratio as an Independent Risk Factor for Mortality in Hospitalized Patients with COVID-19. J. Infect..

[38] Wang C., Deng R., Gou L., Fu Z., Zhang X., Shao F., Wang G., Fu W., Xiao J., Ding X. (2020). Preliminary Study to Identify Severe from Moderate Cases of COVID-19 Using Combined Hematology Parameters. Ann. Transl. Med..

[39] Simadibrata D.M., Pandhita B.A.W., Ananta M.E., Tango T. (2020). Platelet-to-Lymphocyte Ratio, a Novel Biomarker to Predict the Severity of COVID-19 Patients: A Systematic Review and Meta-Analysis. J. Intensive Care Soc..

[40] Doganci S., Ince M.E., Ors N., Yildirim A.K., Sir E., Karabacak K., Eksert S., Ozgurtas T., Tasci C., Dogan D. (2020). A New COVID-19 Prediction Scoring Model for in-Hospital Mortality: Experiences from Turkey, Single Center Retrospective Cohort Analysis. Eur. Rev. Med. Pharmacol. Sci..

